# Application of Alginate-Based Hydrogels in Hemostasis

**DOI:** 10.3390/gels8020109

**Published:** 2022-02-10

**Authors:** Yue Xie, Pan Gao, Fangfang He, Chun Zhang

**Affiliations:** Department of Nephrology, Union Hospital, Tongji Medical College, Huazhong University of Science and Technology, Wuhan 430022, China; xy562443651@126.com (Y.X.); panpangao1121@163.com (P.G.); hefang_105@163.com (F.H.)

**Keywords:** alginate, hydrogel, hemostasis, hemostatic agent, application

## Abstract

Hemorrhage, as a common trauma injury and clinical postoperative complication, may cause serious damage to the body, especially for patients with huge blood loss and coagulation dysfunction. Timely and effective hemostasis and avoidance of bleeding are of great significance for reducing body damage and improving the survival rate and quality of life of patients. Alginate is considered to be an excellent hemostatic polymer-based biomaterial due to its excellent biocompatibility, biodegradability, non-toxicity, non-immunogenicity, easy gelation and easy availability. In recent years, alginate hydrogels have been more and more widely used in the medical field, and a series of hemostatic related products have been developed such as medical dressings, hemostatic needles, transcatheter interventional embolization preparations, microneedles, injectable hydrogels, and hemostatic powders. The development and application prospects are extremely broad. This manuscript reviews the structure, properties and history of alginate, as well as the research progress of alginate hydrogels in clinical applications related to hemostasis. This review also discusses the current limitations and possible future development prospects of alginate hydrogels in hemostatic applications.

## 1. Introduction

Various physical, chemical and human factors may lead to tissue trauma and cause bleeding. Deaths caused by trauma account for about 10% of all deaths worldwide, and blood loss is the most common preventable cause of death after trauma [1]. Hemorrhage and hemorrhagic shock cause 30–40% of traumatic deaths. As such, controlling bleeding in the early stage is a very suitable method to reduce mortality and incidence rate [2,3,4]. Furthermore, perioperative and inspection-related procedures can bring a risk of hemorrhage [4,5,6]. In order to reduce the damage caused by hemorrhage or avoid the occurrence of bleeding, to reduce the use of blood products and improve the survival rate and the quality of life of patients, techniques such as compression or pressing, thermoelectric cauterization, surgical ligation or suture, tourniquet, intravenous administration, and application of topical hemostatic agents have been used to control hemorrhage. The earliest history of treating trauma is the use of hot iron rods by European surgeons to stop bleeding in the 16th century. The history of applying hemostatic materials can be traced back to 1866 [7]. Appropriate hemostatic materials can significantly shorten the bleeding time and reduce the amount of blood products, which is of great significance for trauma or postoperative recovery [8]. The annual cost of complex wound care in North America is $10 billion, and globally, this cost may even exceed $22 billion by 2020, indicating that uncontrollable hemorrhage and wound infections cause a grave economic burden on society [9]. Therefore, the development of rapid, safe, effective and economical hemostatic materials is of great clinical and societal importance.

At present, commercially available hemostatic materials are oxidized cellulose, collagen (Col), gelatin (CE), polyethylene glycol (PEG) polymers, fibrin, thrombin, fibrinogen, cyanoacrylate (CA), porous zeolites, clay minerals, chitosan (CS) and alginate (AG), etc. They can all have a sterling hemostatic effect, however they also have some shortcomings. The carboxyl content of oxidized cellulose ranges from 16% to 24%, and the pH value is about 3.1. Low pH will make it cytotoxic and inactivate many bioactive ingredients, making it limited in its application to sensitive tissues and the combined use of bioactive drugs [10]. Collagen only relies on activating platelets for hemostasis, which is less effective in patients with severe thrombocytopenia and has poor tissue adhesion [11]. Fibrin, thrombin, and fibrinogen, as biologically derivatives, have a short shelf life and risk of disease transmission [12]. Porous zeolite will release large amounts of heat when it absorbs water from the blood, causing thermal damage and necrosis of the surrounding tissues [13,14,15]. Montmorillonite particles have been reported to enter the systemic circulation and cause endothelial damage and distal thrombosis in vital organs, and kaolin hemostatic agents may not be effective for patients with coagulopathy disorders [15,16]. Therefore, researchers have always been intensely interested in developing novel efficient and safe hemostatic materials.

The world has a vast ocean area and abundant ocean resources. With the development and exploration of the ocean, research into and the utilization of ocean resources have received more and more attention in recent years [17,18]. Marine polysaccharides have been widely used in biomedical fields due to their good biocompatibility, biodegradability, non-toxicity, and low prices, such as chitosan, alginate, hyaluronic acid and their derivatives [19,20,21]. Among them, alginate has become a favorite due to its unique advantages in hemostasis. Alginate has safety, biocompatibility, non-immunogenicity, high water absorption and the property of easily forming hydrogels [22,23]. This manuscript will briefly introduce alginate hydrogels, and comprehensively review the research and application progress of alginate hydrogels in medical hemostasis. It also considers the existing challenges, and hopes to contribute to the research on new hemostatic materials of alginate.

## 2. Alginate Hydrogels

### 2.1. Structure, Properties and History

**Figure 1:**
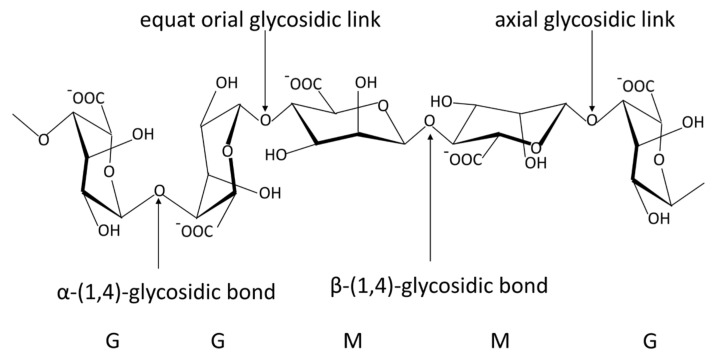
The stylized conformational structure of sodium alginate, including G units, M units and their block patterns and linkages. G, α−L−guluronic acid residue; M, β−D−mannuronic acid residue. The common blocks of G and M units: GG blocks: homopolymer of G units; MM blocks: homopolymer of M units; GM or MG blocks: heteropolymer of G and M or M and G units.

Alginate is a natural anionic polysaccharide, which is a linear chain of β-D-mannuronic acid (M units) and α-L-guluronic acid (G units) linked via 1,4-glycosidic bond, mainly from brown algae and bacteria (Figure 1) [24]. Since the British chemist Standford first extracted alginate from brown algae in 1881, people have conducted extensive research on its physical and chemical properties [25,26]. It has been proven to have excellent biocompatibility, biodegradability and safety [27,28,29]. Alginate has been approved by the FDA as a material generally recognized as safe (GRAS) without special restrictions. Since the 1980s, the applications of alginate have rapidly expanded in food, chemical, printing, agriculture and medicine [24,30,31,32].

**Figure 2:**
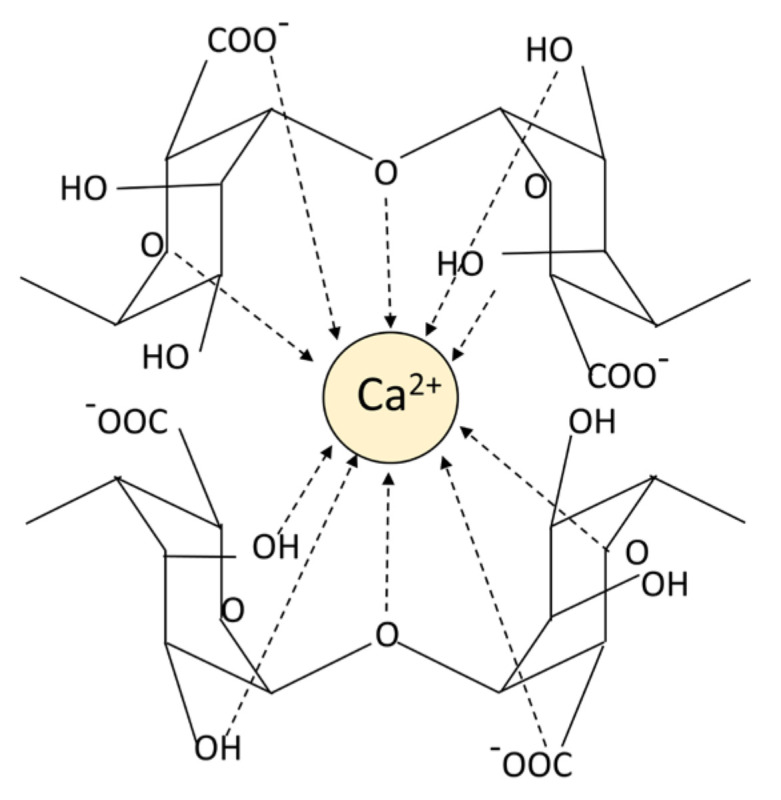
The pattern chart of an egg-box structure.

Alginic acid is insoluble in water, however the sodium alginate forms after binding Na+ can be completely dissolved in water. Hydrophilic sodium alginate can quickly form a hydrogel under extremely mild conditions [33]. In the presence of multivalent cations, the multivalent cations (Ca^2+^ is the most frequent ion) and the monovalent cations (Na^+^ is the most common ion) on the G blocks of alginate undergo an ion exchange reaction, and the G units accumulate to form egg-box conformational hydrogels (Figure 2) [34]. The properties of the hydrogels are related to the M/G composition, the molecular weight of the alginate, source of alginate, the cross-linked ion species and the ionic strength [25,35,36,37]. The rigidity of the alginate polymer chain shows the change of MG < MM < GG, and the increase in molecular weight and ionic strength will lead to the increase in solution viscosity value, which in turn affects the gel properties [25]. G blocks can cross-link with ions to form hydrogels, and high G content can improve the gel strength. Alginic acid can effectively combine various cations to form hydrogels, and the order of binding force is: Mg^2+^ < Mn^2+^ < Ca^2+^ < Sr^2+^ < Ba^2+^ < Cu^2+^ < Pa^2+^ [38,39]. Bacterial-derived alginic acid differs from algal-derived alginic acid in that there are different degrees of acetylated groups linked to D-mannose residues, resulting in better rheology and water absorption, but poor mechanical performance [40]. Due to the existence of bacterial endogenous alginate lyase, the molecular weight of bacterial-derived alginate varies greatly. In addition to production costs and other reasons, bacterial-derived alginate products are not very widespread. Due to its gelling ability, biocompatibility, non-immunogenicity, non-toxicity and safety, alginate has been widely used for hemostasis of various wounds.

### 2.2. Preparation of Alginate Hydrogels

**Figure 3:**
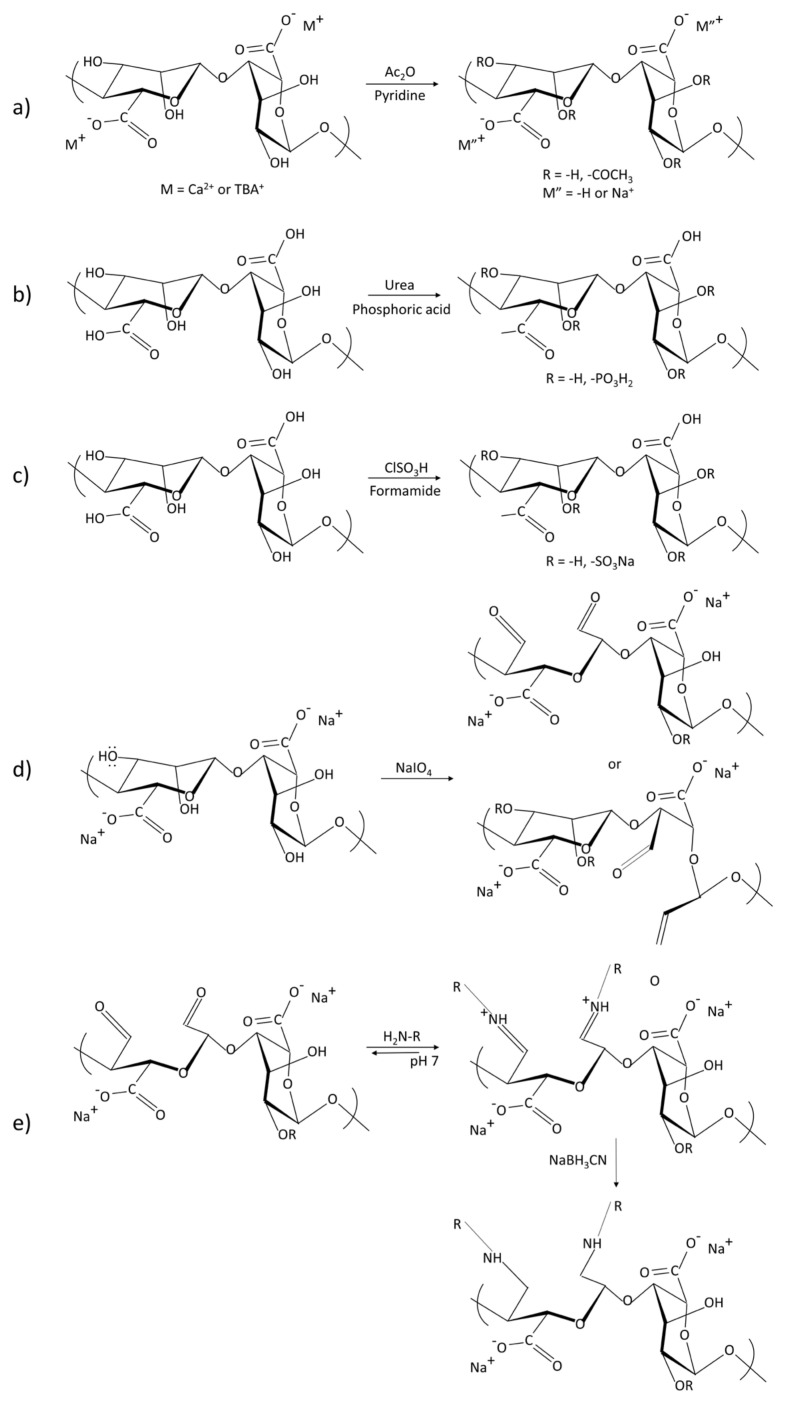
Reaction formula of modified alginate to obtain its derivatives: (**a**) Acetylation of alginate using a mixture of pyridine/acetic anhydride. Gel acetylation: M = Ca^2+^ or TBA^+^. Acetylation of a homogeneous system in DMSO/TBAF, M = TBA^+^, M’ = −H or Na^+^; (**b**) Phosphorylation of alginates; (**c**) Sulfation of alginates. The alginate is sulfated with chlorosulfonic acid; (**d**) Oxidation of sodium alginate; (**e**) Reductive amination of oxidized sodium alginate.

Chemical and physical cross-linking are typical approaches used to form alginate hydrogels (Table 1). The cross-linking methods contain ion interaction, electrostatic interaction, microfluidic technique, divalent chelation, molecular entanglement, self-assemble, guest and host reaction, covalent cross-linking, hydrogen bonds and Schiff cross-linking [41,42]. The gels formed by ionic cross-linking are reversible. The reversibility is why the gels can be degraded in a physiological environment. The gelation rate and the gelation temperature can control gel uniformity and strength when ionic cross-linking [43]. Ionic cross-linking can be carried out by means of diffusion or by controlled triggering (typically the pH or solubility of the ion source) [44,45]. The covalent cross-linking of alginate hydrogels forms a three-dimensional network through copolymerization or polycondensation reaction induced by the cross-linking agent, which is irreversible. The physical properties of hydrogels can be improved by modification. If covalent cross-linking reagents have toxicity, the unreacted chemicals need to be removed thoroughly from gels. Cross-linking density and the reagent type are the main reasons for controlling the mechanical properties of the gels [46]. Chemical modification of alginate hydrogels is also a common means, typically with acetylation, phosphorylation and sulfation of alginate hydroxyl groups, oxidized of alginate and reductive amination of oxidized alginate, esterification and amidation of alginate carboxyl groups, ligation of bioactive ligands with alginate carboxyl groups, and modification of alginate via click chemistry reactions [44,47,48,49,50,51] (Figure 3). Oxidized alginate (OA) or partially oxidized alginate tissue adhesion performance is greatly promoted [52]. There is a continuous progress in the preparation and research of alginate stimuli-responsive hydrogels, such as heat-responsive hydrogels, pH-sensitive hydrogels and light-triggered hydrogels [53,54,55]. A single-component material is usually insufficient to provide enough mechanical properties, biodegradability, adhesion and hemostatic properties. Combined with other materials, alginate composite gels have better properties [56,57]. Then, the alginate gels can be applied to a wider field.

### 2.3. Hemostatic Mechanisms and Advantages of Alginate Hydrogels

Hydrogels are three-dimensional networks formed by cross-linking hydrophilic polymers, and the pores of the network can retain massive water or aqueous solution [69,70]. Hydrogels have become the primary choice of various hemostatic materials as they can simulate the natural cell environment, adapt to soft tissues/hard tissues, and have controllable biological, physical, and chemical properties [71,72]. Ideal hemostatic hydrogels should have fast gelation, good biocompatibility, excellent mechanical properties and sufficient adhesion [73,74]. Alginate hydrogels have gained significant attention in hemostasis, wound healing, and drug delivery as they can quickly form hydrogels under mild conditions and have a high water absorption swelling property, high oxygen permeability, mucosal adhesion property and good biocompatibility [75].

**Figure 4:**
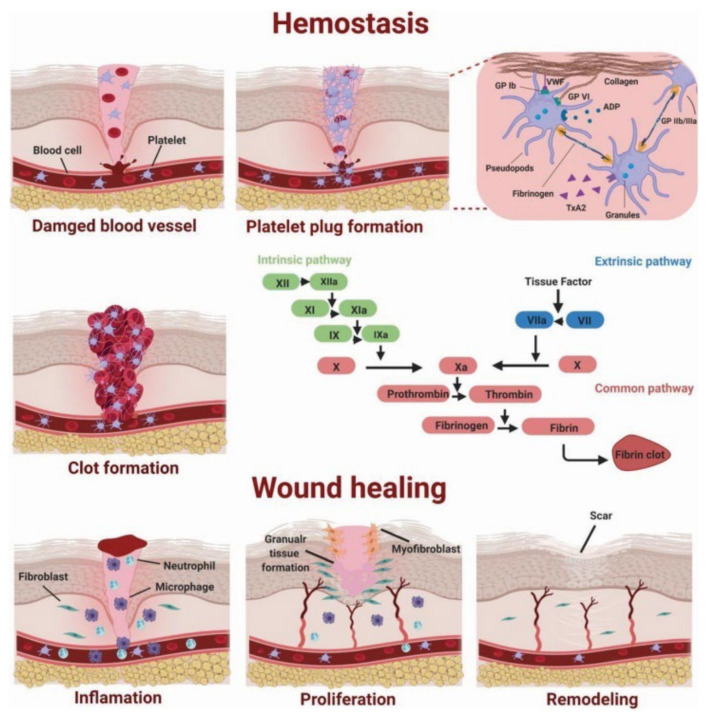
Diagram of the coagulation process. Authorized by John Wiley and Sons Publications, copyright 2020 [80].

The alginate hemostatic materials form hydrogels after absorbing blood or body fluids, adhere to the wound surface, seal capillaries and small blood vessels, and physically compress the bleeding wound to achieve excellent hemostatic effects (Figure 4). Calcium alginate is the most common alginate hemostatic material. Calcium alginate hydrogels can promote the entry of calcium ions into the wound through the ion exchange reaction with sodium ions in the blood. Afterward, they stimulate the production of clotting factors VII, IX, and X, and platelets, activate the coagulation cascade reaction and accelerate the process of hemostasis [76]. Calcium alginate contains phytohemagglutinin, which can aggregate red blood cells and alter erythrocytes morphology, exposing phosphatidylserine on the surface of erythrocytes and accelerating local prothrombin conversion to thrombin [77]. In addition, alginate hydrogels can also be loaded with hemostatic drugs or bioactive ingredients to accelerate hemostasis and healing [78,79]. Alginate, in the form of hydrogels, appears in a series of hemostasis-related products such as medical dressings, hemostatic needles, transcatheter interventional embolization preparations, microneedles, injection agents and hemostatic powders.

## 3. Hemostasis of Superficial Wounds

Alginate materials are highly hygroscopic, moisturizing, and can effectively control hemorrhage and promote wound healing [81,82]. This characteristic is achieved through the formation of hydrogels. In 1962, Winter verified that a moist environment is more appropriate for wound healing than a dry environment [83]. In addition, the alginate fibers confined to the inside of the wound can be removed by biodegradation, without secondary damage to the tissue [84]. Alginate dressings are suitable for wet wounds, and they can play extraordinary roles in hemostasis and healing of superficial wounds.

### 3.1. Fibrous Dressings

**Figure 5:**
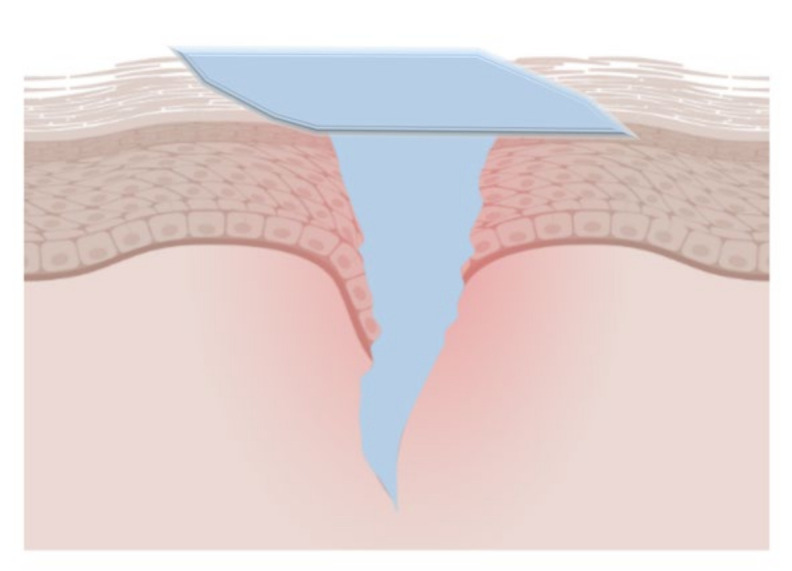
The gel film layer formed by the hydrogel coating materials at the wound site. They can effectively absorb blood and body fluids, seal the wound, and maintain a suitable healing environment.

When fibrous dressings in the dry form make contact with fluid, they can absorb the fluid and form gels, and the hydrogels can maintain a physiologically moist microenvironment and reduce bacterial infection at the wound site (Figure 5). The water absorption rate of pure alginate fiber is 2.2 times its own mass, which is suitable as a hemostatic dressing. Mohandas et al. manufactured alginate hydrogel/nano zinc oxide composite bandages, which had a porosity of 60–70% [85]. The bandages, which are an appropriate hemostatic dressing, can promote blood coagulation and epithelial regeneration. Umar et al. prepared bi-component alginate-hyaluronic acid (AHA) fibers [86]. Compared against commercially available alginate wound dressings (Dimora, marketed by Winner Medical Co., Ltd.), the dressing made of AHA fibers had better liquid absorption capacity and cell adhesion, indicating an overall better performance of the fibers produced by dope mixing. Zhang et al. used a blending method to add nanosilica/hydroxyapatite to alginate dressings, resulting in composites with high porosity [87]. Calcium alginate is common among commercially available dressings, and Ag^+^is usually incorporated to enhance the antibacterial properties of the dressings [88,89,90]. The addition of drug-loaded nanocapsules to alginate dressings using the wet spinning method can improve their mechanical properties and efficiently deliver drugs, favoring wound repair [91].

### 3.2. Films and Membranes

Alginate-based membranes and films are convenient to use and have good hemostatic and wound healing capacities, with water uptake of up to 985% in calcium alginate lyophilized membranes. Forming composite materials with other materials can promote their poor chemical stability and low mechanical strength [56]. Li et al. fabricated the blended alginate sodium/carboxymethyl chitosan membranes cross-linking dual-ion (Sr^2+^and Zn^2+^) (SA/CMCH IPN membranes) through a freeze-drying process [92]. The membrane exhibited excellent water uptake capacity and enhanced ability of cell adhesion, growth factor synthesis and angiogenesis. It is valuable in hemostasis and wound healing. Zhong et al. developed carboxymethyl chitosan/alginate/tranexamic acid composite films, with calcium chloride as the cross-linking agent and glycerin as a plasticizer. The composite membrane had better mechanical and barrier properties, and can slowly release the encapsulated tranexamic acid to accelerate blood coagulation. So, it has shown quite outstanding prospects in the development of hemostatic materials. Hong et al. PVA/HLC/SA hydrogels were prepared by repeated freezing and thawing methods, which were soft and elastic sheets [93]. The hydrogel dressings have excellent effects in swelling, hemostasis, antibacterial and so on. Then they are suitable for hemostasis and healing of traumatic wounds. Abou-Okeil et al. investigated the physical properties and antibacterial properties of hyaluronic acid (HA)/sodium alginate (SA) thin films cross-linked with different metal cations (Ca^2+^, Zn^2+^, Cu^2+^), as well as sulfadiazine and silver nanoparticles effects of incorporation into HA/SA/Ca^2+^ films as bioactive agents [94]. These films have good mechanical properties, water absorption and antibacterial properties, and are suitable as topical bioactive wound dressings. Koga et al. found that aloe/alginate films had good transparency and hemostatic efficiency, and were able to recruit leukocytes and promote wound healing [95]. Shafei used exosomes-loaded alginate hydrogel to study their application at skin wound sites and found that they greatly promoted wound closure, re-epithelialization, and angiogenesis [33]. Du et al. developed a hydrogel material with rapid self-healing and film formation after spraying, which can rapidly gel to form a spray film within 2–21 s [96]. It is a biomaterial suitable for treating large-scale and irregular wounds. Furthermore, sodium alginate can also be used as a hydrophilic coating to enhance the hemostatic ability of composite materials [97].

### 3.3. Hemostatic Sponges

The alginate-based sponges have a porous structure that can quickly absorb a large amount of water, concentrate plasma, stimulate the accumulation of endogenous coagulation factors in the injured area, and form hydrogels as physical barriers to stanch bleeding. They are indicated for large-area hemorrhage wounds and arterial injuries [98]. However, they are improper for uncontrollable and incompressible hemorrhage. Ma et al. prepared nanocomposite sponges of sodium alginate/graphene oxide (GO)/polyvinyl alcohol (SPG) by a freeze-thawing approach and freeze-dried molding [99]. Besides, GO promoted cell proliferation for wound healing and the sponges can load norfloxacin (NFX) to increase its antibacterial capacity. The sponges are suitable for hemostatic materials and wound dressing as a result of appropriate porosity, excellent water absorption and air permeability. Cheng et al. developed oxidized cellulose nanocrystal (TOCN)/alginate (SA) composites, of which the TOCN-30/SA composite sponges had the most efficient hemostatic efficiency [100]. Compared with nitrogen-filled sponges, the porosity, chemical stability and water absorption capacity of this composite sponges are improved, so they are more efficient hemostatic materials. Dowling et al. made hydrophobically-modified (hm) to alginic acid to enhance the adhesion capacity of the formed hydrogels [101]. Compared with the Kerlix™ gauze group, the obtained solid lyophilized sponges have obvious advantages in shortening the clotting time, controlling the amount of bleeding and increasing the clotting effect. This may serve as a safe and effective topical hemostatic agent.

## 4. Hemostasis of Vessel and Viscera

### 4.1. Hemostatic Needles

Hemorrhage after puncture of biological tissue with syringe needles is largely inevitable, and bleeding after puncture may lead to serious consequences [5]. The hemorrhage can be well controlled by hydrogel-coated hemostatic needles, which seal punctured tissue by a solid-to-gel phase transition in situ [102].

**Figure 6:**
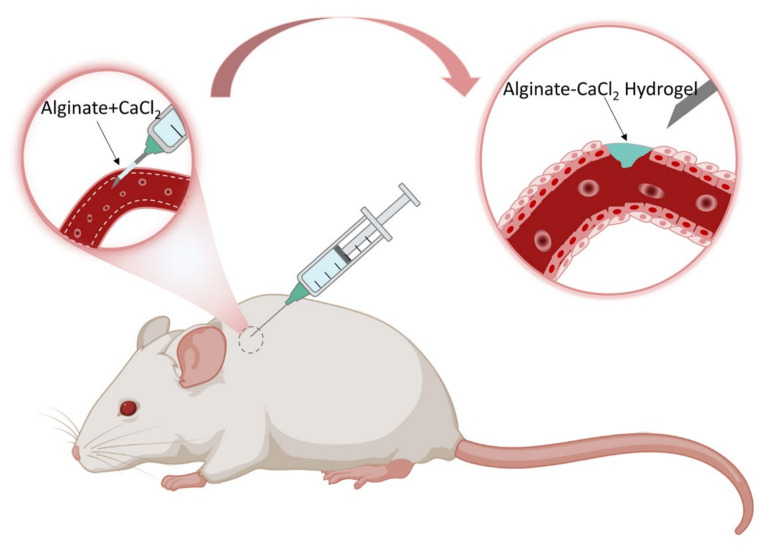
The illustration exhibits how the hemostatic needles work through the coating. Their syringe needles are topically coated with cross-linking Alginate-CaCl2 hydrogel film coatings, which can swell to form hydrogels during puncture and play a significant role in preventing hemorrhage after puncture.

**Figure 7:**
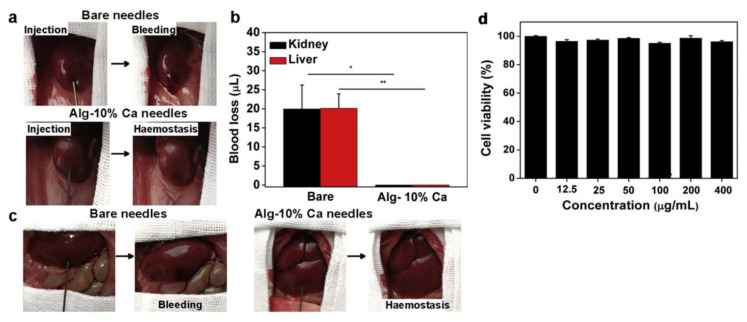
The haemostatic capability of the Alg-Ca-coated needles in viscera puncture. (**a**) Photographs showing the haemostatic effect produced by Alg-Ca-coated needle (22 G) in rat kidney: bare needle (top) and Alg-Ca-coated needle (bottom). (**b**) Quantitative analysis of blood loss from rat kidney (*n* = 8, * *p* < 0.05) and liver (*n* = 8, ** *p* < 0.01). (**c**) Photographs showing the haemostatic effect of Alg-Ca-coated needle (22 G) in rat liver: bare needle (**left**) and Alg-Ca-coated needle (**right**). (**d**) Cytocompatibility evaluation of the Alg-Ca. [103] Copyright 2020 Elsevier Ltd.

Ren et al. developed hemostatic needles coatings with alginate-CaCl_2_-based hydrogels, which can effectively prevent hemorrhage following tissue puncture (Figure 6 and Figure 7) [103]. The dry-formed hydrogels coatings on the needles can be converted into hydrogels upon hydration, and the adhesion of the hydrogels to the wound would be increased so that the hydrogels were separated from the needles when the needles were withdrawn and sealed the wound in situ. The coatings prepared by Alg-10%Ca precursor solutions had sterling uniformity and prominent mechanical properties. The evaluation of the adhesive strength of the coatings through the stainless-steel plates and porcine skin verified that the solid coatings will not fall off before puncture, whereas the hydrogels would adhere to the tissue after swelling. The results of hemostasis evaluation indicated that the needles with Alg-10%Ca coating exhibited universal synchronous hemostasis ability in venous blood vessels and internal organs. Alginate hydrogels coated needles can prevent bleeding after tissue puncture easily, rapidly and effectively, and have a promising application in injection and sampling. Inspired by this, Wei et al. secured a hydrogel made of sodium alginate, hyaluronic acid, and calcium carbonate [104]. The dried hydrogel coating can quickly reabsorb the aqueous solution to form a physical embolus to seal the puncture tract, resulting in effectively preventing blood loss. CD34 antibody within the hydrogel attracted progenitor cells to accelerate puncture tract healing. The hydrogel coating of the needle can act as a carrier for drugs to assist hemostasis and puncture wound healing by delivering drugs. Xu et al. also made a hydrogel composed of sodium alginate, hyaluronic acid, and calcium carbonate [105]. The hydrogel-coated needle successfully prevented the puncture and bleeding of AVF and AVG in rats. The hydrogel may be used to improve hemorrhage after AVF or AVG cannulation. Besides, Yin et al. synthesized gelatin–tannic acid composite hydrogels with high viscosity to achieve hemostasis in arterial puncture [106]. The excellent viscosity, mechanical strength and burst pressure of the hydrogels facilitated in situ hemostasis for arterial puncture. The hydrogels could produce hemostatic needles for different vessels.

### 4.2. Embolic Materials

Nowadays, minimally invasive transcatheter arterial embolization (TAE) is the best non-laparotomy option for rupture and bleeding of solid internal organs [107]. It is widely used in the treatment of cancer, aneurysm, parenchymal organ hemorrhage, gastrointestinal hemorrhage and postpartum hemorrhage [108,109,110]. The first documented arterial embolization operation was performed by Dawbain et al. in 1904 [111]. Standing embolic agents can cause chronic inflammation and even tissue damage. Gelatin particles are suitable for embolization hemostasis due to their non-antigenicity, good biocompatibility and degradability; however they cannot control the embolization precision well. The exploitation of novel and appropriate embolic agents is very important for the development of TAE [112,113,114]. In recent years, calcium alginate hydrogels have become a research hotspot of TAE embolization materials [115]. Many investigations of TAE have demonstrated the feasibility and effectiveness of TAE for hemostasis in visceral trauma or rupture and vascular embolization [116,117,118,119]. The alginate-based embolic agents can also cut off the blood supply of the tumor tissue and release the drug gradually at the embolization site, which are developed as dual-effect drugs for tumor embolization and chemotherapy [114,120]. Furthermore, they can be used in preoperative preparation for surgical procedures to prevent intraoperative bleeding. Becker et al. used the two-way injection method (PHG alginate, 2.5 wt%, via venous injection and CaCl_2_·2H_2_O, 0.68 M, via arterial injection) to test the biocompatibility and embolic properties of the calcium alginate hydrogels in rat kidney capsule and rabbit kidney, in determining their suitability as embolic materials [121]. Calcium alginate hydrogel was rapidly gelled in situ, acted as a stable polymer stopper to prevent all flow out of the kidney during the study, and had good biocompatibility. However, this two-way injection requires rich clinical experience and meticulous operation, which limits its use.

**Figure 8:**
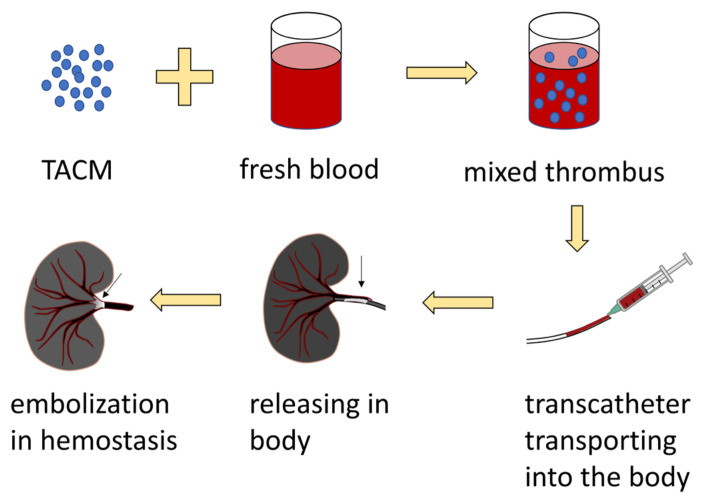
The preparation and application process of TACMs.

Sodium alginate microsphere vascular embolization agents (KMG) are widely used in interventional embolization therapy as a result of the following advantages: (1) They have good mechanical stability and biocompatibility, and do not cause chemical or immune reactions after embolization; (2) The final degradation products are polysaccharides that do not participate in the metabolic circulation of the human body; (3) They are quickly swell and are incarcerated after absorbing water, which effectively reduces accidental embolization; (4) They can embolize terminal arterioles. Rong et al. secured thrombin-loaded alginate-calcium microspheres (TACMs) by electrostatic droplet technology (Figure 8) [122,123]. TACMs mixed thrombus showed a significant increase in thrombin strength compared to the human own thrombus. The thrombin loaded in TACMs further shortens the clotting time. In the rabbit renal embolism model, bleeding completely stopped within 1.5 ± 1 min without embolic material reflux, and the survival rate of experimental animals was 100% in one week. It demonstrated that TACMs mixed thrombus had a good efficacy for transcatheter embolization of solid internal organ rupture and hemorrhage, and had clinical transformation potential. Research on alginate microspheres loaded with other drugs may develop more ideal embolic hemostatic products.

## 5. Hemostasis of Deep and Irregular Wounds

### 5.1. Injectable Hydrogels

**Figure 9:**
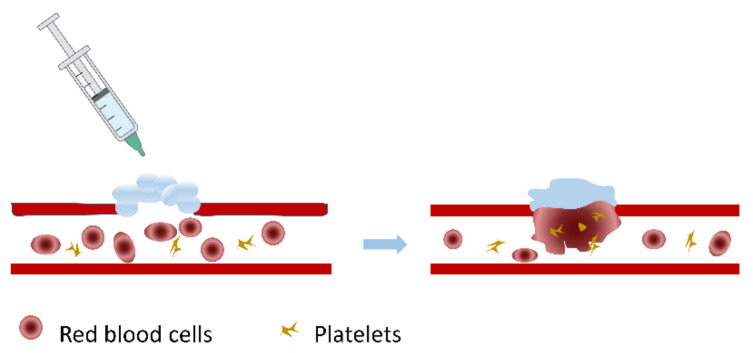
Schematic diagram of the action of injectable hydrogels at the bleeding site. Injectable hydrogels in the form of solutions gel in situ at the wound site under certain conditions, recruit red blood cells and platelets, promote blood coagulation, and achieve the purpose of hemostasis.

The injectable hydrogels offer excellent features and enable the complete filling of deep and irregularly shaped wounds with hemorrhages, thereby forming effective mechanical barriers and reducing pain and scars (Figure 9). Injectable hydrogels can also conveniently and efficiently target delivery of encapsulated drugs to designated sites to assist in hemostasis or other therapeutic effects.

**Figure 10:**
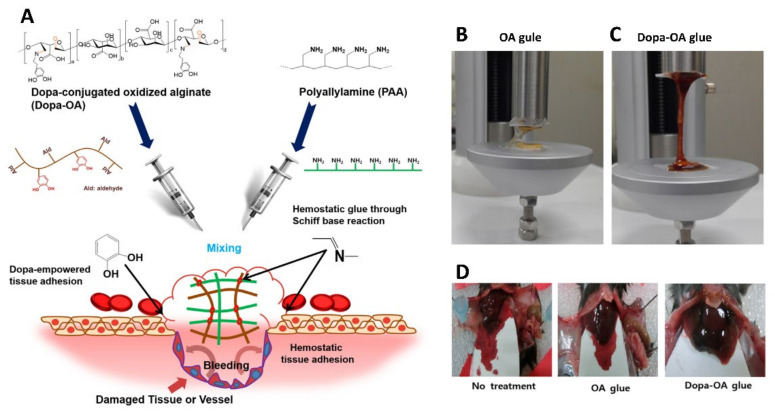
(**A**) The chemistry behind the formation of Dopa-OA hydrogel glue. (**B**,**C**) The Dopa-OA hydrogel displayed higher tensile strength than OA glue. (**D**) Hemostatic performance of Dopa-OA glue with respect to OA glue and the control, when used in a mouse liver injury model. Reproduced with permission [128]. Copyright 2019, Springer Nature.

Zhai et al. fabricated a supramolecular hydrogel, the co-assembly of a cell adhesive peptide conjugate (Pept-1) and alginate (ALG), to control bleeding and promote wound healing [124]. Compared with Pept-1 hydrogel, the mechanical properties, hemostatic properties and wound healing capabilities of Pept-1/ALG hydrogel were greatly improved. In the hemostasis experiment in vitro, Pept-1/ALG hydrogel decreased the clotting time to 41 s, which was almost 28-fold faster than the control. In vivo hemostasis experiment, the blood loss in the Pept-1/ALG hydrogel treatment group was approximately 18% of that in the untreated control group. The alginate hydrogels are very promising for co-assembly with other biologically active ingredients to construct nanocomposite structures for hemostasis. Zhang et al. developed injectable in situ self-cross-linking hydrogels, made of sodium alginate, hemoglobin and carbon quantum dots (SA@Hb@CQDs) [125]. The hydrogels are versatile for rapid hemostasis, real-time monitoring, and chemokinetic therapy (CDT). In addition, the hydrogels are also antibacterial, promote wound healing and prevent tumor recurrence, which can be used for postoperative adjuvant therapy of cancer. Song et al. formed tissue-adhesive hemostatic hydrogels [126]. Alginate was selected as a primary polymer, which was oxidized for the Schiff base forming alginate and encoded with dopa. Dopa-OA hydrogels had high stiffness and elasticity, which can be used as a tissue glue for hemostasis. Oxidized sodium alginate improved the adhesion of the hydrogels to a certain extent [127]. Dopamine, as a mussel protein derivative material, can greatly improve the tissue adhesion ability of alginate hydrogels [61,128,129] (Figure 10). Kong et al. synthesized an injectable self-healing hydrogel containing CuS nanoparticles (CuS-NPs) based on N-carboxyethyl chitosan and oxidized sodium alginate [130]. This hemostatic material had good results in hemostasis, antibacterial and healing promotion, and due to its excellent self-healing and injectability, it can adapt well to the shape of the wound.

### 5.2. Microspheres

Microspheres are commonly prepared by spray drying, extrusion, and emulsification/gelation method, and, due to the large surface area and small volume, microspheres can fully contact the wound and enter narrow and small regions. Microspheres can produce micro/nano effects to promote blood absorption and cell adhesion, resulting in rapid hemostasis (within a few seconds). However, when encountering a large blood flow, they are easily washed away, so it is necessary to modify the raw materials and choose materials with special functions or composite materials to improve the efficiency of hemostasis.

Shi et al. developed composite microspheres made of carboxymethyl chitosan, sodium alginate, and collagen (CSCM), which can facilitate hemostasis [131]. Compared with the commercially available hemostatic agent (CMPHP), CSCM microspheres exhibited deeper pores and higher surface roughness, resulting in more platelets aggregation and adhesion, and have better hemostatic properties. CSCM may be useful in controlling hemorrhage in military and civilian emergencies. Huang et al. obtained silk fibroin (SF)/sodium alginate (SA) microspheres by emulsifying cross-linking [132]. The rough surface of SF/SA microspheres increased the cell adhesion ability. When the volume ratio of SA: SF was 2:1, the sample microspheres had the roughest surface and gathered the largest number of RBCs, leading to the fastest blood clotting rate. SF is natural fibrin, which can effectively improve the tissue adhesion of SA hydrogels. At the same time, SA can improve spheroidization and promote coagulation. SF/SA2 microspheres have the potential to be developed as a new type of hemostatic powder. Jia et al. and Zhang et al., respectively, prepared berberine-coated alginate-based composite microspheres [133,134]. The coated berberine enhanced the antibacterial capacity, water absorption capacity and biodegradability of the materials. The experimental groups with higher berberine content, such as SCC-10B (7%) and BACG-6B (6%), showed significantly enhanced hemostatic properties, indicating that these composite polysaccharides microspheres have potential value for hemostatic applications. Adhesive self-healing hydrogels can also be used for incompressible hemostasis, such as bone bleeding [135]. The Alginate-Based Composites obtained by Huang et al. combined the characteristics of sodium alginate (SA) and carboxymethyl chitosan (CMC) resulting in a better hemostatic effect than single-component SA or CMC, and good bioadhesive and degradable properties [136]. Wu et al. synthesized microspheres with a porous chitosan core and a compact calcium alginate shell layer [76]. The electrostatic adsorption of the chitosan core and the Ca^2+^ of calcium alginate worked together to achieve better hemostasis. They are suitable for severe bleeding in civil and war situations.

### 5.3. Hydrogel Beads

Simple instillation is a common method for producing alginate hydrogel beads, which can easily prepare gel particles encapsulated with various components and then be developed as hemostatic powders [137,138]. Liu et al. secured a hydrogel bead made of tissue factor (TF), collagen and alginate (TCA), which could play a role of good composite hemostatic [79]. Alginate, as the matrix of TAC beads, made contributions to entrapping TF-liposomes and collagen, absorbing water to form a hydrogel and controlling the release of TF. In vitro coagulation experiments, TCA beads, collagen/alginate beads, TF-liposomes/alginate beads, alginate beads and the control of recalcified blood alone required 4.5, 8.6, 6.2, 12.3 and 14.4 min to achieve coagulation, indicating TAC beads are a kind of potential hemostatic agent. Fathi et al. synthesized zeolite-loaded alginate-chitosan beads by a two-phase synthesis method, which targeted multiple mechanisms to improve hemostasis [139]. The electrostatic properties of chitosan enhanced the erythrocyte adhesion of beads. The porous structure of zeolite increased blood absorption and concentrated clotting factors. The hydrophilicity of alginate led to blood absorption and retention while playing a role as a physical barrier. The 4A4Z1C and 4A8Z1C beads induced coagulation within 15 s (14.4 ± 0.31 s and 14.76 ± 1.36 s). Due to their rapid hemostatic ability, the composite beads are expected to be used for traumatic bleeding control.

### 5.4. Microneedles

Microneedles (MNs) are a novel class of transdermal drug delivery systems (TDDS) with advantages such as pain relief, ease of application, low infection risk, controlled drug release, and efficient delivery [140]. The development of microneedles with hydrogel materials can be used for the sustained release of drugs and minimally invasive examination, which is beneficial as they reduce the occurrence of traumatic bleeding [141,142]. Microneedles or microneedle patches made of alginate composite materials can be used for sustained-release transdermal delivery of insulin, reducing the issues such as high bleeding probability, painful sensation, inconvenient life, and low compliance of diabetic patients due to multiple daily injections [142,143,144]. MNs can be used for cancer treatment, vaccine delivery, blood pressure control, etc. [145,146,147]. Percutaneous immunization of MNs has less risk of inducing inflammation and skin damage than intradermal injection, and is less likely to cause severe bleeding and complications. The implantable degradable microneedle patches can simultaneously have the excellent ability of rapid intraoperative hemostasis and long-term postoperative chemotherapy [148]. MNs also have great application space in minimally invasive examinations, such as examining or monitoring cytokines, glucose, cholesterol, etc. [149,150,151].

## 6. Conclusions and Future Perspectives

For people with acute massive blood loss or suffering from coagulation disorders, the body’s inherent hemostatic mechanism cannot meet the needs of timely hemostasis, and the existence of suitable hemostatic materials and technologies is very important. How to prevent the occurrence of bleeding events, stop bleeding in time and improve prognosis is of great clinical and social significance at present. The alginate-based hydrogels have biocompatibility, safety, water absorption, moisture retention and biodegradability, so they are well suited for application as hemostatic materials. Different types of alginate materials are suitable for hemostasis or prevention of bleeding in different situations (Table 2).

However, insufficient cell adhesion occurs due to a large number of negative charges on the surface of alginate materials and the lack of biochemical clumps that can adhere to cells. Alginate hydrogels mainly rely on divalent cations to be formed through physical cross-linking, and are sensitive to monovalent cations, citrate ions, chelating agents and phosphate ions, resulting in a construct that is not stable enough. The mechanical properties of sodium alginate after swelling are not ideal. Alginate hydrogel alone is not fast enough in hemostasis, and it is impossible to monitor wound healing. Through continuous exploration of chemical modification, composite components, changing the mode of application and loading drugs, alginate hydrogels can be improved in the mechanical properties, adhesion, stability, and hemostatic performance, thereby enabling alginate hydrogels to play a timelier and more effective role in hemostasis. In the future, low-cost, multi-function, composite combination, targeted delivery, in situ hemostasis with wound formation, loading of biologically active ingredients, and intelligent monitoring of wound healing may become the research direction of sodium alginate hydrogels.

## Figures and Tables

**Table 1:** Preparation method of alginate hydrogels.

Type of Reaction	Materials	Methods Used	Form of Gels	Properties	Ref.
Ionic cross-linking	Alginate + ZnO	Casting and solvent evaporation	Bi-layered hydrogel films	Antibacterial properties, promotes healing capacity	[58]
Ionic cross-linking	Alginate + shellac	Microfluidic technology	Amphiphilic hydrogel–solid dimer particles	Amphiphilic structure, biocompatibility	[45]
Ionic cross-linking	AuNP-CaCO_3_ + sodium alginate	/	Hydrogel precipitation	Fast response, pH-response, and ultrahigh sensitivity	[59]
Electrostatic interaction and divalent chelation	Alginate + N-carboxymethyl chitosan	/	Dual-crosslinked hydrogels	Tunable mechanical properties, efficient wound closure	[60]
Hydrogen bonds and Schiff cross-linking	Oxidized sodium alginate + dopamine	/	Hydrogels	Self-healing, high tensile strength and stretchability	[61]
Molecular entanglements	Alginate + carboxymethylcellulose sodium + chitosan	Thermoforming, high-speed blending	Composite hydrogels	Water vapor permeability	[62]
Molecular entanglements	Alginate + PVA + PVP	Freezing-thawing method	Hydrogels	Bioadhesive strength and mechanical properties	[63]
Self-assemble	Alginate + copolymer F127	/	Hydrogels	Thermo-responsive behavior	[64]
Guest and host reaction	PEG-Adamantane + β-CD + alginate	/	Hydrogels	Self-healing	[65]
Photo-cross-linking	Alginate + PVP + chitosan	Gamma-radiation	Hydrogel pads	Photoreactivity, hygroscopicity	[66]
Ionically cross-linking, covalent cross-linking	laponite + PVA + alginate		Nanohybrid hydrogels	Blood coagulation	[67]
Covalent cross-linking, ionically cross-linking	OA + ECM + Amine-rGO		Double-network hydrogel	Improved mechanical properties and electrical conductivity	[68]

**Table 2:** Summary of Different Types of Alginate Materials.

Type	Materials	Active Ingredients	Properties	Indications	Ref.
Fibrous dressings	Alginate + nZnO	nZnO	High porosity, antibacterial properties	Severe bleeding and wounds at risk of infection	[85]
	Alginate + chitosan + hyaluronic acid	/	High swelling absorption properties	Moist wound care	[86]
Films and membranes	Sodium alginate (SA) + carboxymethyl chitosan (CMCH)	Sr^2+^, Zn^2+^	Cell adhesion enhancement and antibacterial properties	Various stages of wound healing	[92]
	Hyaluronic acid (HA) + sodium alginate (SA)	Sulfadiazine, AgNPs	Mechanical property enhancement, antibacterial properties	Local wound hemostasis and care	[94]
	Gel-ADH+ SA-mCHO	/	Rapid spray-filming performance	Rapid and massive hemostasis	[96]
Sponges	Sodium alginate (SA) + graphene oxide (GO) + polyvinyl alcohol (SPG)	Norfloxacin (NFX)	High water uptake and gas permeability	Hemostasis in superficial wounds, Wound dressings	[99]
	Oxidized cellulose nanocrystals (TOCN) + Sodium alginate (SA)	/	High chemical stability and water absorption	Rapid local hemostasis	[100]
	Hm-alginate	/	High adhesiveness	Dressing for hemostasis	[101]
Hemostatic needles	Alginate + CaCl_2_	/	Coating, hemostasis in situ	Prevention of bleeding after vascular and tissue puncture	[103]
	SA + HA + calcium carbonate	CD34	Coating, hemostasis in situ	Stab wound hemostasis and healing	[104]
	SA + HA + calcium carbonate	/	Coating, hemostasis in situ	Hemorrhage after AVF or AVG cannulation	[105]
Embolic materials	Alginate + CaCl_2_·2H2O	/	Minimal trauma and good hemostatic effect	Hemostasis in solid visceral organ rupture and hemorrhage	[121]
	Alginate + CaCl_2_	Thrombin	Embolic hemostasis	Parenchymal visceral hemorrhage	[123]
	CaCO_3_-Alginate	/	High safety and availability	Hepatocellular carcinoma therapy	[152]
Injectable Hydrogels	Pept-1 + ALG	Pept-1	High mechanical strength and hemostatic efficiency	Hemostasis in noncompressible wounds and irregular wounds	[124]
	Oxidized sodium alginate (OA) + dopamine	Dopamine	High stiffness and elasticity	Hemostasis in deep tissue	[126]
	N-carboxyethyl chitosan + oxidized sodium alginate	CuS-NPs	Injectability and self-healing	Hemostasis of in situ wounds	[130]
Hemostatic powders	Carboxymethyl chitosan + sodium alginate + collagen	/	High surface roughness and good biodegradability	Hemostasis in emergency conditions	[153]
	Alginate + silk fibroin	/	High surface roughness	Rapid hemostasis in vitro and in vivo	[132]
	Alginate + collagen + TF liposomes	Tissue factor (TF)	Efficient and inexpensive, adaptation to various shapes of wounds	Rapid hemostasis in traumatic injury and deep wounds	[79]
Microneedles	Alginate + maltose + CaCl_2_	Insulin	Excellent mechanical strength and toughness, great swelling and dissolution properties	Sustained release transdermal delivery of insulin	[154]
	Sodium alginate + Bacillus Calmette–Guérin (BCG)	Bacillus Calmette–Guérin (BCG)	Small risk of inducing inflammatory response and skin damage	Transcutaneous immunization with vaccine	[146]

## Data Availability

All data is included in the manuscript.
